# Ti_3_C_2_ (MXene) nanosheets disrupt spermatogenesis in male mice mediated by the ATM/p53 signaling pathway

**DOI:** 10.1186/s13062-023-00382-w

**Published:** 2023-06-13

**Authors:** Yang Wei, Ruilin Bao, Le Hu, Yanqing Geng, Xuemei Chen, Yixian Wen, Yingxiong Wang, Mao Qin, Yue Zhang, Xueqing Liu

**Affiliations:** 1 Joint International Research Laboratory of Reproduction and Development, School of Public Health, Chongqing Medical University, Chongqing, People’s Republic of China; 2 Department of Obstetrics and Gynecology, The First Hospital of Lanzhou University, Key Laboratory of Gynecologic Oncology of Gansu Province, Lanzhou, People’s Republic of China; 3 College of Basic Medicine, Chongqing Medical University, Chongqing, People’s Republic of China; 4 Department of Andrology, Women and Children’s Hospital of Chongqing Medical University, Chongqing, People’s Republic of China; 5 Department of Obstetrics and Gynecology, Women and Children’s Hospital of Chongqing Medical University, Chongqing, People’s Republic of China

**Keywords:** Spermatogenesis, Ti_3_C_2_ nanosheets, Spermatogonia, DNA damage, ATM/p53 signaling pathway

## Abstract

**Background:**

Two-dimensional ultrathin Ti_3_C_2_ nanosheets are increasingly being used in biomedical applications owing to their special physicochemical properties. But, the biological effects of its exposure on the reproductive system is still unclear. This study evaluated the reproductive toxicity of Ti_3_C_2_ nanosheets in the testes.

**Results:**

Ti_3_C_2_ nanosheets at doses of 2.5 mg/kg bw and 5 mg/kg bw in mice caused defects in spermatogenic function, and we also clarified an underlying molecular mechanism of it in vivo and in vitro model. Ti_3_C_2_ nanosheets induced an increase of reactive oxygen species (ROS) in testicular and GC-1 cells, which in turn led to the imbalance in oxidative and antioxidant systems (also known as oxidative stress). Additionally, oxidative stress often induces cellular DNA strand damages via the oxidative DNA damages, which triggered cell cycle arrest in the G1/G0 phase, leading to cell proliferation inhibition and irreversible apoptosis. ATM/p53 signaling manifest key role in DNA damage repair (DDR), and we demonstrate that ATM/p53 signaling was activated, and mediated the toxic damage process caused by Ti_3_C_2_ nanosheet exposure.

**Conclusion:**

Ti_3_C_2_ nanosheet-induced disruption of proliferation and apoptosis of spermatogonia perturbed normal spermatogenic function that was mediated by ATM/p53 signaling pathway. Our findings shed more light on the mechanisms of male reproductive toxicity induced by Ti_3_C_2_ nanosheets.

**Graphical abstract:**

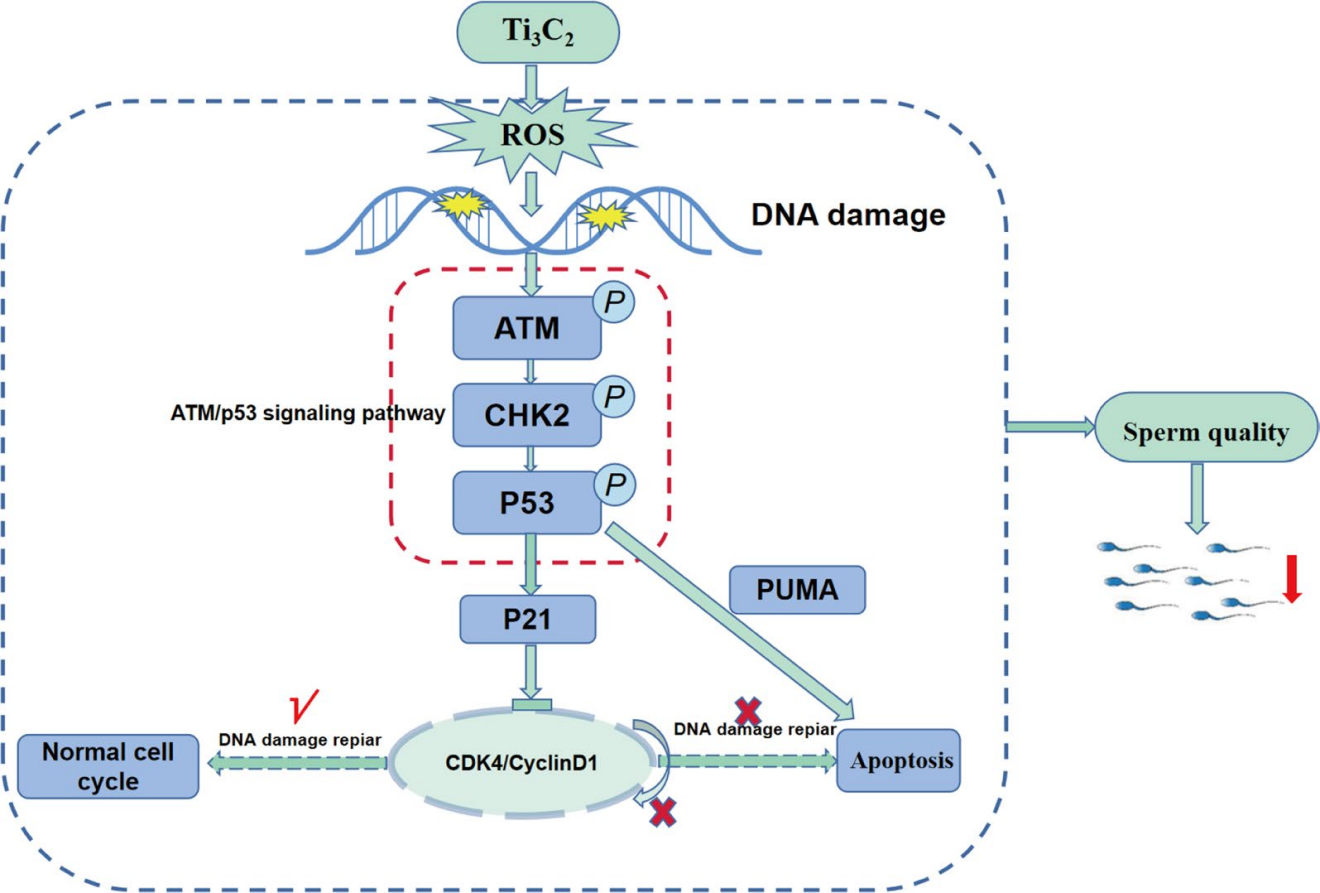

## Introduction

With an increasing decline in global fertility, reproductive health has gained the spotlight in medical research. It is estimated that 20–30% of infertility cases are caused by men [1]. Epidemiological studies have shown that sperm quality and quantity have shown a downward trend [2], with male toxicity caused by environmental pollutants being an important factor. Exposure to environmental pollutants results in changes in gene expression, hormonal imbalances, reproductive disorders, and sperm abnormalities [3]. Environmental pollutants are divided into chemical toxins, physical factors, and biological toxins. Importantly, chemical toxins are the most common and have the most profound impact. Many studies have focused on the effects of endocrine-disrupting chemicals (EDCs), pesticides, and heavy metals on the reproductive system. However, an increasing relevance of nanomaterials to human beings (cosmetics, paints, medical products, textiles, personal care, and other products) warrants the verification of their biosafety as they pose potentially high risks to human health [4]. Nanomaterials refer to materials with nanorange size, which is generally 1–100 nm. In the medical field, nanomaterials can be applied in drug transportation and controlled release due to its noticeable features such as increased permeability enabling crossing through biological barriers and improved biocompatibility. These particular properties of nanomaterial suggest it can be utilized in cancer therapeutics [5]. On the other side, its excellent performance can also have adverse effects on human health. For instance, ZnO nanoparticles (NPs) are well known and widely used as catalysts in clothing, paints, and cosmetic products. ZnO NPs have the potential to induce apoptosis in testicular cells through DNA damage caused by reactive oxygen species, with possible adverse consequences for spermatogenesis and therefore, male fertility [6]. Lanthanum oxide (La_2_O_3_) NPs are widely used in catalysis, and water treatment, La_2_O_3_ NPs induce abnormal spermatogenesis in mice via Nrf-2/ARE signaling [7].

MXenes is a new type of two-dimensional (2D) transition metal carbides or nitrides [8]. The general formula of MXene is M_n+1_X_n_T_x_ (n = 1–3), where M represents an early transition metal (such as Ti, Zr, Hf, or Ta), X refers to carbon and/or nitrogen, and T represents surface terminal groups (such as oxygen, hydroxyl, chlorine, or fluorine). It was derived from the corresponding MAX phase by the selective etching of the interlayer Al atoms of the transition metal carbide, Ti_3_AlC_2_ [9]. The 2D layered structured material with unique surface termination and properties have showed great potential in biomedical applications such as photothermal therapy [10, 11], diagnostic imaging [12], antimicrobial formulations [13, 14], biosensing [15, 16], and drug delivery [17, 18]. But, some negative impacts have also followed. Graphene is the earliest discovered 2D nanomaterial, oxidized Graphene at low concentrations increase reactive oxygen species (ROS) production and induce autophagy in human neuroblastoma cell lines SK-N-BE(2) and SH-SY5Y [19]. Recently, it has been shown that exposure to Ti_3_C_2_ (MXene) nanosheets, a kind of new 2D nanomaterial, leads to their accumulation in the lungs, thereby interfering with respiratory function through the downregulation of SP-B levels in alveolar epithelial cells [20]. Abnormal placental development and altered placental lipid metabolism following exposure to Ti_3_C_2_ nanosheets cause neurodevelopmental disorders in offspring mice [21]. Previously, we found that Ti_3_C_2_ nanosheet exposure inhibited the growth of villus explants and impaired the function of HTR-8/SVneo cells [22]. However, experimental studies on the biosafety of Ti_3_C_2_ nanosheets are limited, and the effects of Ti_3_C_2_ nanosheets on the male reproductive system have not been studied yet.

Previous studies have shown that the biological effects of NPs are closely associated with DNA damage. Human DNA is damaged by endogenous and exogenous factors tens of thousands of times per day [23]. Recent investigations have indicated that exposure to cobalt nanoparticles causes DNA damage in A549 cells, and exposure to nickel nanoparticles causes DNA damage in BEAS-2B cells [24, 25]. DNA damage response (DDR) system in vivo play an important role in repairing gene variation and maintaining the stability of cell genome. Activated ATM directly or indirectly phosphorylates a wide scope of targets, including p53 and H2AX, to inhibit DDR [25, 26]. Acute oral administration of nano-calcium hydroxide particles causes DNA damage induction by generating free radicals and altering the expression levels of *p53* gene and proinflammatory cytokines [27]. Nanoparticles inhibit spermatogenesis by inducing cell cycle arrest and inhibiting proliferation in the testes [28]. Studies have equally demonstrated that oxidative stress can lead to testicular damage and apoptosis in rats, resulting in reduced sperm quality [29]. Oxidative stress is a key factor in many diseases causing cell damage, and the generation of ROS is associated with DNA damage, membrane lipid peroxidation, and apoptosis. Studies have shown that environmental toxicant-induced male reproductive toxicity is associated with increased oxidative stress and reduced antioxidant capacity in the testes [30, 31].

In this study, we investigated the effects of Ti_3_C_2_ nanosheet exposure on spermatogenesis and its potential mechanisms. Kunming male mice and GC-1spd cell lines were exposed to different doses of Ti_3_C_2_ nanosheets, and the underlying mechanisms were investigated. Our findings complement those from the study of the biosafety of Ti_3_C_2_ nanosheet exposure as well as provide clinical applications of this nanomaterial.

## Materials and methods

### Synthesis and characterization of Ti_3_C_2_ nanosheets

Following earlier techniques [32]. Ti_3_C_2_ nanosheets were created by etching Ti_3_AlC_2_ in an HCl/LiF solution. In a nutshell, 4 g of LiF and 2 g of Ti_3_AlC_2_ powder were slowly added to 80 mL of HCl (9 M) and stirred for 24 h at 35 °C. The compound was washed with distilled water numerous times until the pH of the supernatant reached 6. After being sonicated for 1 h under an Ar flow, the MXene powder was recovered using centrifugation at 3500 rpm for 1 h. Transmission electron microscopy (TEM, JEM-2100FEG, JEOL, Japan), scanning electron microscopy (SEM, JSM 6701 F, JEOL, Japan), atomic force microscopy (AFM, Multimode 8, Bruker, USA), and X-ray photoelectron spectroscopy (XPS, ESCALAB 250Xi, Termo Fisher Scientifc, USA) were all used to analyze the ultrastructure and morphology of the nanosheets.

### Animal model

In this study, male mice aged 8 weeks (KM-strain, 28–30 g) were acquired from the lab animal center of Chongqing Medical University (Certificate: SICXK (YU) 2007-0001). The Institutional Animal Care and Use Committee of Chongqing Medical University (Chongqing, China) gave its approval to all animal experiments. They were given unlimited access to food and water, kept in a stable environment with a relative humidity of 55% ± 10%, and kept at 22 °C ± 2 °C with a 12-hour light/dark cycle.

Based on research on Ti_3_C_2_ nanosheets as a new photothermal agent for cancer therapy, we selected the lowest reported dose as the highest dose (5 mg/kg) per week in the present study [33]. After being adapted to the environment for 1 week, 60 male mice were randomly assigned to four groups of 15 mice each, three experimental groups were exposed to Ti_3_C_2_ nanosheets (1.25, 2.5, and 5 mg/kg body weight) through intravenous injections once weekly for 4 weeks, and the control group were injected with saline. Ti_3_C_2_ nanosheets were dispersed in saline and had good dispersion. The concentrations of Ti_3_C_2_ nanosheets in saline were 0.25 mg/mL, 0.5 mg/mL and 1 mg/mL, respectively. The volume of the Ti_3_C_2_ nanosheet suspension used for exposure was 0.05 mL/10 g body weight. All mice were sacrificed 7 days after exposure to Ti_3_C_2_ nanosheets, and the testes and epididymis were sampled for analyses.

### ICP-MS analysis

To track the Ti_3_C_2_ nanosheets’ biodistribution, testicular tissue was gathered. Ti_3_C_2_ nanosheet accumulation in the testes was carried out by Beijing Zhongkebaice Technology Co., Ltd. Using inductively coupled plasma mass spectrometry, the (Ti) content was quantified (ICP-MS, 7800, Agilent Technologies Inc., USA).

### Sperm quality evaluation

The left epididymis was utilized to calculate the rate of sperm malformation, while the right epididymis was removed for the sperm count test. In order to fully release the sperm, the epididymis was divided into pieces and placed in 1 mL of normal saline that had been preheated to 37 °C. 10 µL of the sperm suspension was placed on a hemocytometer, and the number of sperm was counted under a microscope. The suspension was placed onto a slide and gently pushed in order to test for sperm deformity. Slides were dried, methanol-fixed, and eosin-stained. Finally, under a microscope, abnormal sperms were counted. During morphological analyses, at least 500 sperm cells per animal were counted to assure precision.

### Testicular histopathology

Fresh mouse testes were excised, preserved for 4–6 h at 25–37 °C in 4% paraformaldehyde, and then dehydrated with a gradient series of alcohol before being embedded in paraffin (melting point, 60 °C). Hematoxylin and eosin (H&E) staining was done on the testicular Sect. (5 μm thick), and the results were seen under a microscope (BX40, Olympus, Tokyo, Japan).

### Immunohistochemistry

Immunohistochemistry was used to identify the corresponding antigens. After being deparaffinized in xylene and heated at 60 °C for one hour, the tissue sections were rehydrated using an alcohol gradient series. The endogenous peroxidase activation was suppressed for 10 min after being incubated with 1% H_2_O_2_ following the 15 min boiling of sodium citrate buffer to extract the antigens. The next step was blocking with 10% goat serum for 15 min at 25 °C. The sections were then treated with secondary antibodies (goat anti-rabbit IgG, 1:1000 dilution, Santa Cruz Biotechnology Inc., CA, USA) for 30 min. at 37 °C after being exposed to primary antibodies, such as Ki67 (1:200, Abcam Inc., Cambridge, MA, USA), at 4 °C overnight. The sections were then stained with 3,3-diaminobenzidine for 3 min and hematoxylin for 2 min after three PBS rinses. They were then observed and photographed under an Olympus microscope.

### Immunofluorescence (IF)

Mouse testicular tissue sections were dewaxed and hydrated, and the repair antigens were prepared. After three PBS washes, testicular tissue and GC-1 cell slices were blocked with 10% goat serum in 5% bovine serum albumin. They were then treated with primary antibodies against Ki67 and γ-H2AX (1:200, Cell Signaling Technology, Danvers, MA, USA) for an overnight period at 4 °C. The sections were incubated with Dylight 488-conjugated affinity purified goat anti-rabbit IgG (1:200, Abcam Inc., Cambridge, MA, USA) for 1 h in the dark at 37 °C the next day after being washed three times with PBS. The nuclei were then stained with DAPI, and the pictures were captured on camera and examined under a microscope.

### TUNEL staining

To detect apoptotic germ cells in the testes of the mice, a Colorimetric TUNEL Apoptosis Assay Kit (Beyotime, China) was utilized. Tissue sections were deparaffinized in xylene, heated for 1 h at 60 °C, and rehydrated using an alcohol gradient series. Proteinase K solution (20 µg/mL) was applied to the sections for 15–30 min at 20–37 °C. The tissue sections were rinsed three times with PBS before being incubated in 3% hydrogen peroxide and cleaned with PBS. Biotin labeling solution (50 µL) was applied to the sections and incubated for 1 h at 37 °C, light shielded. The slices were incubated at room temperature for 30 min after being washed three times in PBS with 50 mL streptavidin-HRP working fluid. They were then stained with 3,3-diaminobenzidine and hematoxylin, observed and photographed under a microscope.

### Cell culture and treatment

Mouse spermatogonia GC-1 cell line was obtained from the Concorde Cell Bank. GC-1 cells were grown in high-glucose DMEM supplemented with 10% fetal bovine serum (Gibco, Waltham, MA, USA), 100 U**/**mL penicillin, and 100 µg/mL streptomycin. Cell lines were incubated at a constant temperature 37 °C and 5% CO_2_ in an incubator. GC-1 cells were exposed to Ti_3_C_2_ nanosheets (0, 6, 12.5, 25, and 50 µg/mL) for 24 h. Control cells were treated with saline solution.

### Transmission electron microscopy (TEM)

After being exposed to various doses of Ti_3_C_2_ nanosheets for 24 h, GC-1 cells were washed in PBS and digested with trypsin solution. The supernatant from the centrifugation of the cell suspensions at 1200 rpm for 10 min was discarded. In order to evaluate the samples, the acquired cells were pre-fixed with 2.5% glutaraldehyde at the College of Life Sciences of Chongqing Medical University.

### **Assessment of ROS, malondialdehyde (MDA), superoxide dismutase (SOD) levels**

Testicular tissues of the mice were collected 28 days after Ti_3_C_2_ nanosheets treatment. Mouse testicular tissue was homogenized (tissue: normal saline = 1:9), and the supernatant was collected. Levels of oxidative stress-related factors, including ROS, MDA, and SOD, were quantified using enzyme-linked immunosorbent assay (ELISA) kits.

2,7-Dichlorodihydrofluorescein diacetate (DCFH-DA), a fluorescent probe, was used to measure intracellular ROS levels (Beyotime, China). Ti_3_C_2_ nanosheets at varying concentrations were applied to GC-1 cells for 24 h. 200 µL of 10 µmol/L DCFH-DA (DCFH-DA: DMEM = 1:1,000) were added after the medium was aspirated, and the mixture was then incubated at 37 °C for 30 min. Using a microplate reader with excitation and emission settings at 485 and 530 nm, respectively, the DCFH fluorescence of the cells was detected. With the aid of the appropriate kits, the MDA and SOD levels were determined (Beyotime, China). Additionally, cells were taken out of the culture dishes and lysed for 5 min. The lysate was then centrifuged at 12,000 rpm for 5 min at 4 °C. According to the manufacturer’s instructions, the supernatant was collected and the intracellular levels of MDA and SOD were found.

### Comet assay

Following a 24-h exposure to Ti_3_C_2_ nanosheets, GC-1 cells were collected. Then, 10 µL of the cell suspension was added to a low melting point agarose (LMPA) solution, transferred to glass slides that were covered in a normal melting point agarose (NMPA), and a third layer of LMPA agarose was added to plug any remaining holes in the second agarose layer. Slides were soaked in lysis buffer for two hours at 4 °C after the agaroses had solidified. At 25 V, electrophoresis was carried out for 20 min. The slides were exposed to neutralization buffer for 10 min after electrophoresis before being stained with ethidium bromide. Finally, photos were captured on camera and viewed under a microscope.

### Flow cytometry analysis

An Annexin V-FITC Apoptosis Detection Kit was used for flow cytometry analysis (E-CK-A212; Elabscience, Wuhan, China). The cells were digested with trypsin before being transferred to 1.5 mL tubes for further investigations. The cells were placed in 1 mL of PBS buffer and centrifuged at 1000 rpm for 5 min, yielding approximately 1 × 10^6^ cells. Following two PBS washes, 100 µL of binding buffer was added and mixed. Flow cytometry was used to detect apoptosis using Annexin V-FITC (5 µL) and PI (5 µL).

GC-1 cells were collected and washed twice with PBS before being resuspended in 0.1 mL of PBS. To avoid aggregation, 0.9 mL of ice-cold 75% ethanol was injected dropwise on a vortex. After all samples were stored in the dark at room temperature for 30 min, the prepared working fluid (RNase A: PI = 1:9) was added for cell cycle analysis by flow cytometry.

### Cell viability assay

Cell viability was determined using the manufacturer’s cell fluorescence counting kit (CCK-F) (C2013S; Beyotime, Jiangsu, China). GC-1 cells were grown in BeyogoldTM all-black 96-well plates at a density of 5 × 10^3^ cells/well for 24 h. The cells were subsequently exposed to various concentrations of Ti_3_C_2_ nanosheets and saltwater. After adding 100 µL of calcein AM detection solution to each well and incubating the mixture at 37 °C for 30 min, the fluorescence intensity was measured using fluorescence microscopy. Finally, fluorescence intensity was used to calculate cell viability.

### Alignment of RNA-seq data and differential expression analysis

The transcriptome sequencing analysis of the testicular tissue samples was performed by Nanjing Personal gene technology CO., LTD (Nanjing, China). The main methods of genome sequencing are total RNA extracted, purified, and libraries built. Followed by Next-Generation Sequencing, an Illumina-based sequencing platform that performs double-end sequencing of these libraries. The DESeq2 software was used to identify differentially expressed genes (DEGs), |log2 Fold Change| > 1, *P* < 0.05, were considered different. The GO stats tool was used to perform functional enrichment for KEGG.

### Western blotting analysis

Testicular tissues and GC-1 cells were lysed in RIPA buffer containing protease inhibitor from a protein extraction kit (Beyotime, China), and the BCA protein assay (Beyotime, China) was used to measure the protein concentration. Proteins were separated using 12% SDS-PAGE and transferred to polyvinylidene fluoride (PVDF) membranes. The membranes were blocked for 1 h at 37 °C with 5% dried fat-free milk in PBS-Tween 0.1% (PBST), and were incubated with specific primary antibodies against γ-H2AX (1:5,000), Cleaved Caspase3, p21, *p*-p53, *p*-CHK2, ATM (1:1,000, Cell Signaling Technology, Danvers, MA, USA), Bax, Bcl-2, CyclinD1 and CDK4 (1:500, Wanleibio, Shenyang, China), PCNA (1:5,000, Abcam Inc., Cambridge, MA, USA), PUMA, *p*-ATM, CHK2 (1:1000, BOSTER, Wuhan, China), p53, and β-actin (1:1,000, ZSGB-BIO, Beijing, China) overnight at 4 °C. The next day, the secondary antibodies (1:1,000, BOSTER, Wuhan, China) were incubated for 1 h at 37 °C with anti-mouse or anti-rabbit HRP-conjugated antibodies. Protein bands were detected using enhanced chemiluminescence (NCM Biotech, Suzhou, China), and the densities of those bands were quantified by ImageJ software.

### **Statistical analysis**

GraphPad Prism software (version 8.0) (Graph-Pad Software Inc., La Jolla, CA, USA) was used for the data analysis. Student’s *t*-test was used to analyze two-group comparisons, and ANOVA was used to compare more than two groups. *P* < 0.05 was considered significant. All variables were expressed as mean ± standard deviation (SD).

## Results

### Ti_3_C_2_ nanosheets can enter testicular tissue and GC-1 cells

**Fig. 1:**
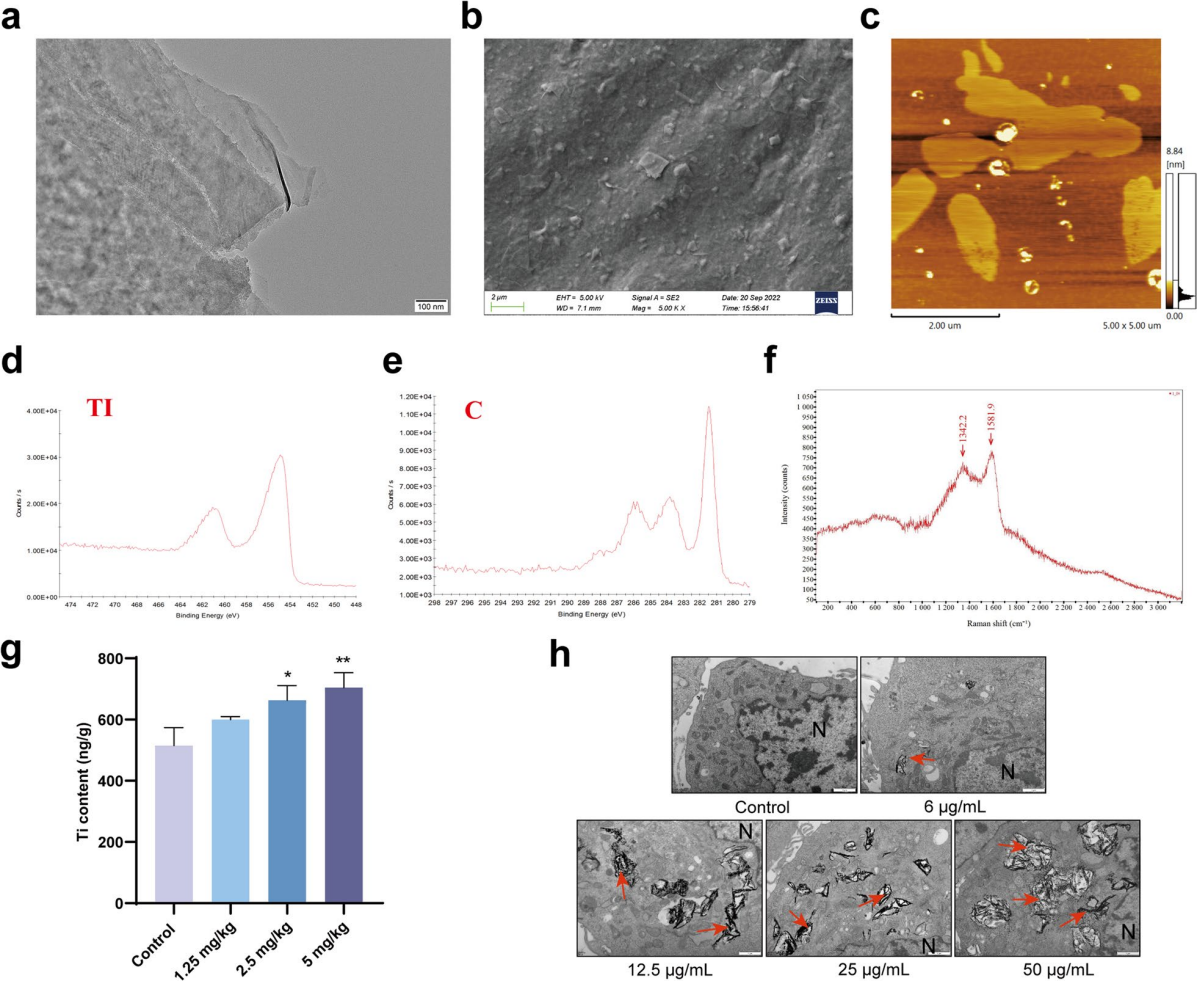
Characterization of Ti_3_C_2_ nanosheets and distribution of Ti_3_C_2_ nanosheets. **a** TEM image of single-layer Ti_3_C_2_ nanosheets. **b** SEM image of Ti_3_C_2_ nanosheets. **c** AFM image of Ti_3_C_2_ nanosheets. **d**–**e** XPS spectrum of Ti_3_C_2_ nanosheets in the Ti and C core-level regions. **f** Raman spectrum of Ti_3_C_2_ nanosheets. **g** Ti content was measured in testis by ICP-MS. **h** Distribution of Ti_3_C_2_ nanosheets and cellular ultrastructure of GC-1 were observed by TEM (Scale bar = 1 μm). Red arrows indicate Ti_3_C_2_ nanosheets. N: nucleus. All data were expressed as means ± standard deviation (n = 3). *P < 0.05, **P < 0.01, compared with the control group

The morphology of the Ti_3_C_2_ nanosheets was evaluated using TEM and SEM; the lateral size of the ultrathin nanosheet structure was 0.05-1 μm (Fig. 1a, b). The thickness of the well-dispersed nanosheets, determined through AFM analysis, was 1–4 nm (Fig. 1c). In addition, the chemical state and elemental composition were analyzed using XPS (Fig. 1d, e), showing characteristic peaks in the region of the Ti and C core layers. The Raman spectra of the Ti_3_C_2_ nanosheets exhibited two peaks at approximately 1342.2 and 1581.9 cm^− 1^, implying that the Ti_3_AlC_2_ phase was completely converted into Ti_3_C_2_ nanosheets (Fig. 1f). ICP-MS analysis was performed to measure the biodistribution of Ti in testes. The Ti content was significantly increased (*P* < 0.05) in the testes compared to that in the control group after intravenous injection of Ti_3_C_2_ nanosheets in the 2.5 and 5 mg/kg group (Fig. 1g). TEM was used to observe the distribution of the nanosheets and the ultrastructural variation in GC-1 cells treated with Ti_3_C_2_ nanosheets. Images of treated GC-1 cells clearly demonstrate the cellular uptake of the nanosheets. All Ti_3_C_2_ nanosheets were distributed mainly within the cytoplasm of GC-1 cells (Fig. 1h).

### Ti_3_C_2_ nanosheets exposure reduces sperm count in mouse

**Fig. 2:**
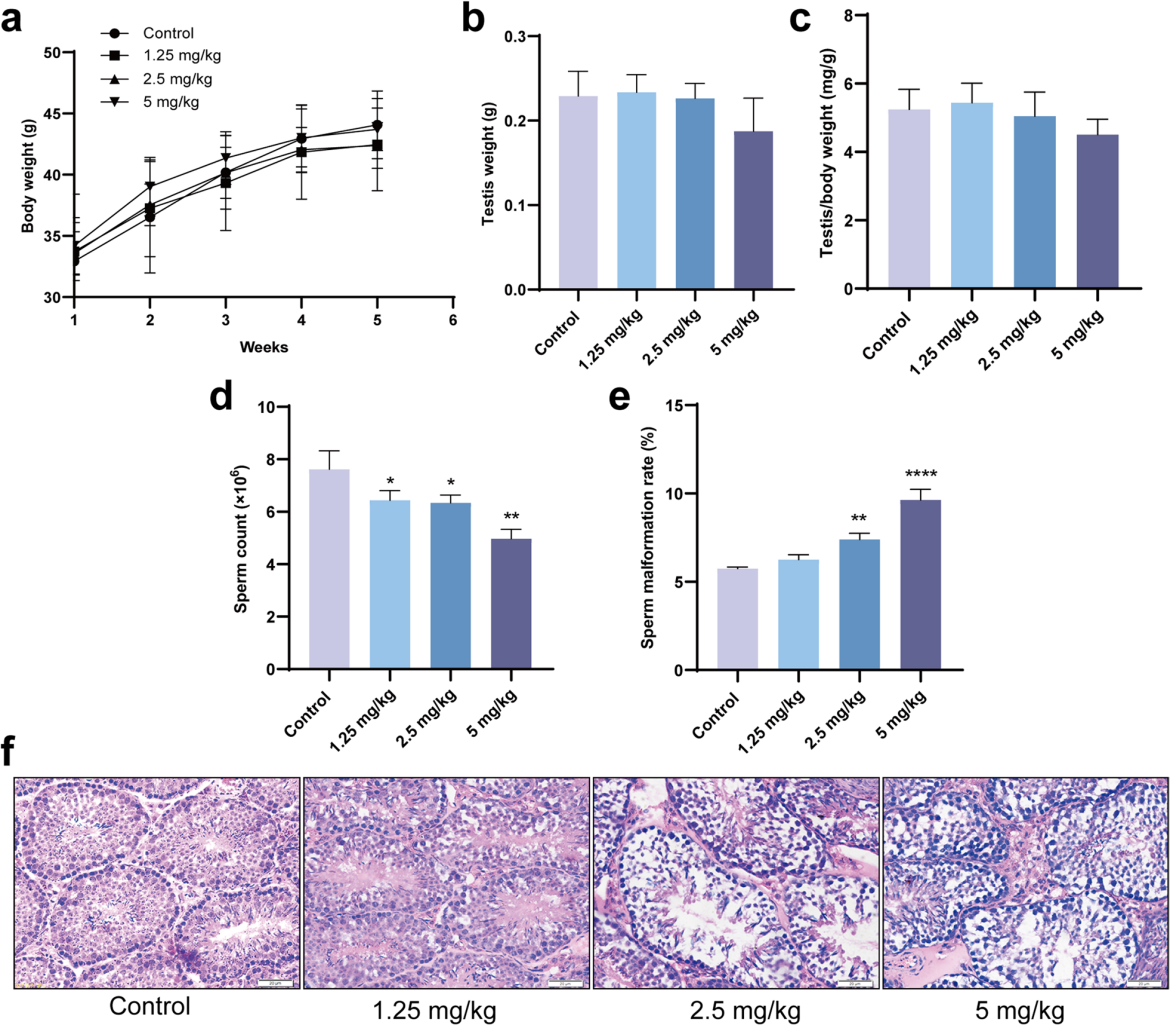
Body weight and testicular weight, epididymal sperm count and malformation rate, testicular H&E staining. **a** The body weight of mice after exposure to Ti_3_C_2_ nanosheets at different doses. **b** The testis weight of mice at different doses. **c** The organ coefficient of the testis at different doses. **d** The sperm count in the epididymis. **e** The sperm malformation rate in the epididymis. **f** Testicular sections were stained with H&E for the histopathological assessment. All data were expressed as means ± standard deviation (n = 5). *P < 0.05, **P < 0.01, ****P < 0.0001, compared with the control group

The effect of Ti_3_C_2_ nanosheet exposure on sperm quality was also evaluated. There was no significant change in body weight and organ coefficient of the testis between Ti_3_C_2_ nanosheet-exposed mice and controls (Fig. 2a–c). The epididymal sperm number was considerably lower in Ti_3_C_2_ nanosheet-exposed mice than that in the controls (Fig. 2d). The epididymal sperm malformation rate was remarkably higher in the 2.5 and 5 mg/kg groups than that in the controls (Fig. 2e). We examined pathological changes in the testes using hematoxylin and eosin (H&E) staining after Ti_3_C_2_ nanosheets exposure. Mice in the control group showed normal seminiferous tubules and germinal cell arrangement. In the 2.5 and 5 mg/kg groups, H&E staining showed vacuoles and disorganization of the germ cell layers in the seminiferous tubules (Fig. 2f).

### Ti_3_C_2_ nanosheets exposure induces oxidative stress in mouse testes and GC-1 cells

**Fig. 3:**
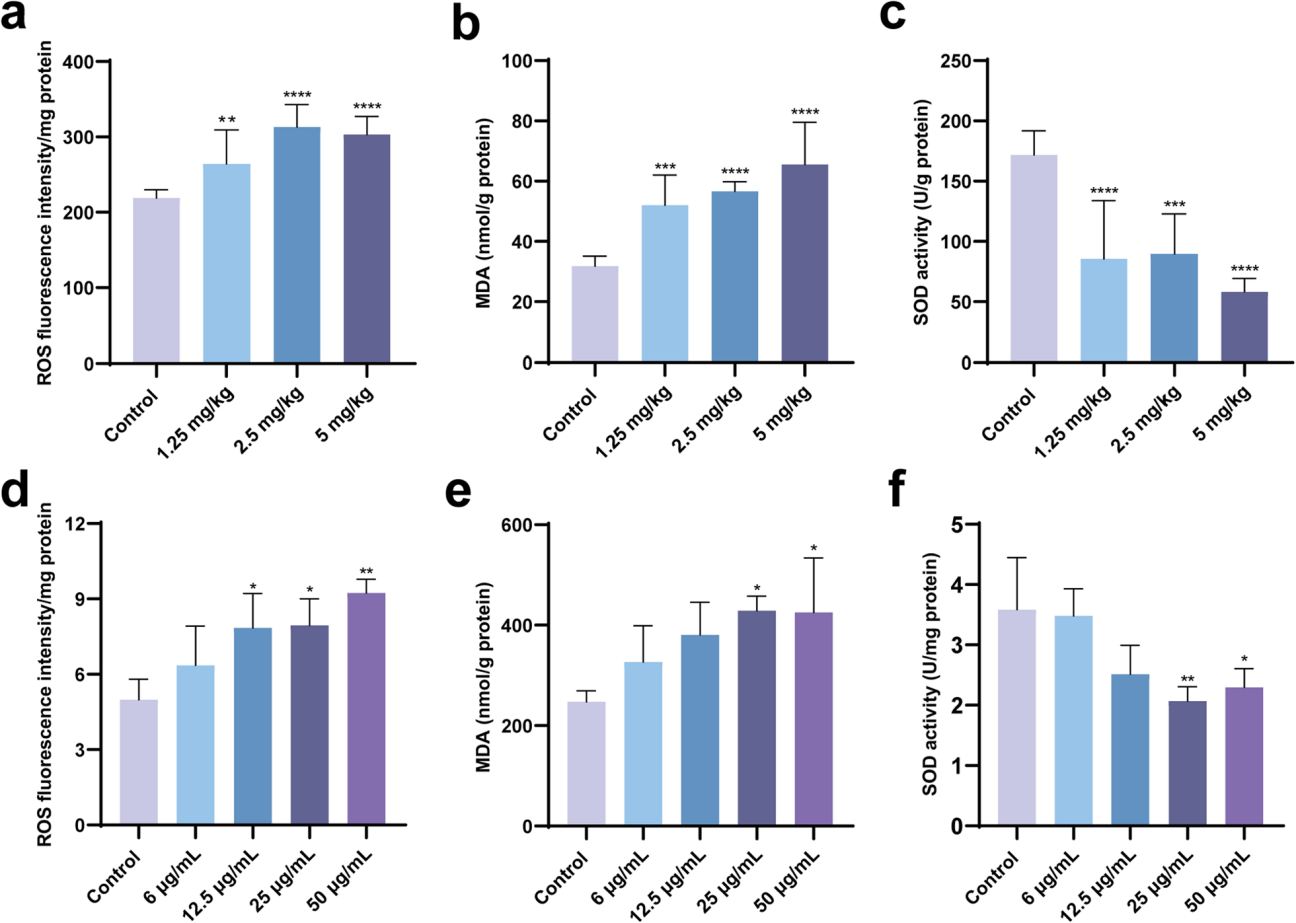
Ti_3_C_2_ nanosheets exposure raised the level of oxidative stress in mouse testes and GC-1 cells. ROS (**a**), MDA (**b**), SOD (**c**) levels in testes were determined from testis tissues. **d** Intracellular ROS were examined at 24 h after Ti_3_C_2_ nanosheets exposure. Then the contents of MDA (**e**) and the enzyme activities of SOD (**f**) were determined. All data were expressed as means ± standard deviation (n = 3). *P < 0.05, **P < 0.01, ***P < 0.001, ****P < 0.0001, compared with the control group

The oxidative stress levels were measured using ELISA kits and oxidation-antioxidation assay kits to explore the effect of Ti_3_C_2_ nanosheets on oxidative stress in mouse testes and GC-1 cells. The ROS levels and MDA content in Ti_3_C_2_ nanosheets-treated mice groups were significantly higher than those in the control group. The SOD activity in Ti_3_C_2_ nanosheets-treated mice groups were significantly lower than those in the control group (Fig. 3a–c). In vitro, the intracellular ROS levels of GC-1 cells in the Ti_3_C_2_ nanosheet-treated groups (12.5, 25, and 50 µg/mL groups) were remarkably higher than those in the control group. The MDA content in the Ti_3_C_2_ nanosheet-treated groups (25 and 50 µg/mL groups) was remarkably higher than that in the control group. The SOD activity in the Ti_3_C_2_ nanosheet-treated groups (the 25 and 50 µg/mL groups) was significantly lower than that in the control group (Fig. 3d–f). These findings indicate that Ti_3_C_2_ nanosheets induced oxidative stress in mice testes and GC-1 cells.

### Ti_3_C_2_ nanosheets exposure causes DNA damage in mouse testes and GC-1 cells

**Fig. 4:**
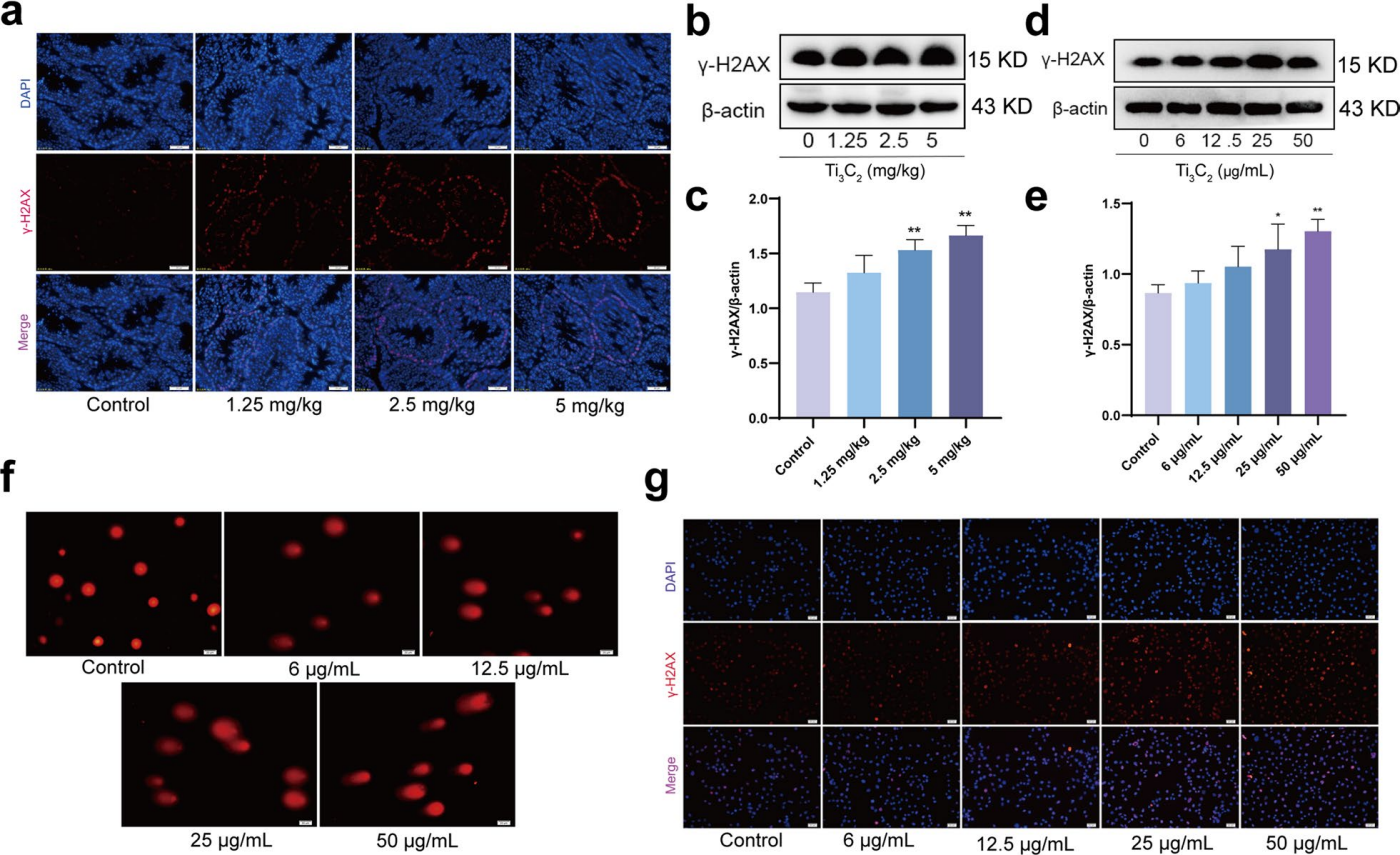
Ti_3_C_2_ nanosheets exposure causes DNA damage in mouse testes and GC-1 cells. **a** DNA damage was shown via staining of the seminiferous tubules with the DSB marker γ-H2AX (red). Magnification at 400 ×. **b**, **c** Testicular γ-H2AX was detected using Western blotting. **d**, **e** The γ-H2AX protein level in GC-1 cells were detected using Western blotting. **f** The typical comet tails of GC-1 cells at different doses. Magnification at 200 ×. **g** Representative images of γ-H2AX (red puncta) detected by immunofluorescence (γ-H2AX, DAPI, Merge, Magnification at 100 ×). All data were expressed as means ± standard deviation (n = 3). *P < 0.05, **P < 0.01, compared with the control group

To determine the effect of Ti_3_C_2_ nanosheets on DNA damage in testicular tissues and GC-1 cells, γ-H2AX (a sensitive marker of DNA damage) was assessed through IF staining of testicular sections. As shown in Fig. 4a, many spermatogonia stained positively in the spermatogenic tubules of the testes after treatment with Ti_3_C_2_ nanosheets. The protein expression level of γ-H2AX was significantly elevated in Ti_3_C_2_ nanosheet-exposed mouse testes and GC-1 cells (Fig. 4b–e). In addition, DNA damage was also observed in Ti_3_C_2_ nanosheet-exposed GC-1 cells, as assessed by the comet assay (Fig. 4f). As shown in Fig. 4g, γ-H2AX-positive cells were significantly enhanced in the Ti_3_C_2_ nanosheet-exposed GC-1 cells. These findings strongly indicate that Ti_3_C_2_ nanosheet exposure induces DNA damage in the mouse testes and GC-1 cells.

### Ti_3_C_2_ nanosheets exposure inhibits proliferation in mouse testes and GC-1 cells

**Fig. 5:**
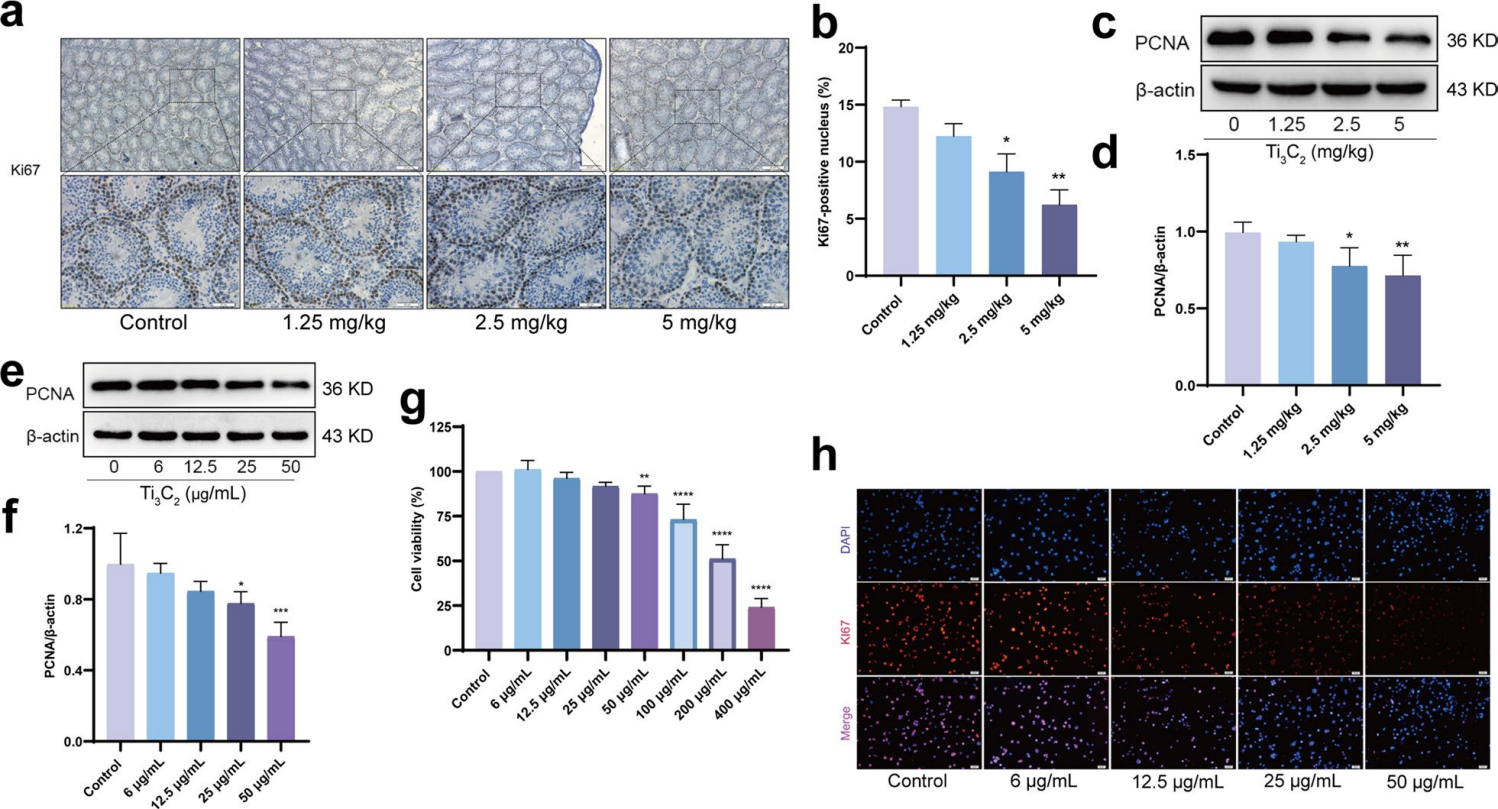
Ti_3_C_2_ nanosheets exposure inhibits proliferation in mouse testes and GC-1 cells. **a**, **b** Cell proliferation was examined by using Ki67 staining. Ki67^+^ cells were counted. Magnification at 100 × and 400 ×. **c**, **d** Testicular PCNA was detected using Western blotting. **e**, **f** Cellular PCNA was detected using Western blotting. **g** The viability of GC-1 cells was examined via the using CCK-F assay. **h** GC-1 cells were stained with Ki67 by immunofluorescence. (γ-H2AX, DAPI, Merge, Magnification at 100 ×). All data were expressed as means ± standard deviation (n = 3). *P < 0.05, **P < 0.01, ***P < 0.001, ****P < 0.0001, compared with the control group

Ki67 immunohistochemical staining was used to measure the proliferation of mouse testicular germ cells to explore the effect of the nanosheets on spermatogonia proliferation. The proportion of positive Ki67 stained nuclei in the Ti_3_C_2_ nanosheet-exposed mouse testes was significantly than that in the control group (Fig. 5a, b). The expression level of PCNA, an additional marker of cell proliferation, was dramatically decreased in the testes of Ti_3_C_2_ nanosheet-exposed mice (Fig. 5c, d). Next, we studied the effect of the Ti_3_C_2_ nanosheets on the proliferation of GC-1 cells, as well as the protein expression levels of PCNA (Fig. 5e, f). Cell viability decreased in Ti_3_C_2_ nanosheet-exposed GC-1 cells in a dose-dependent manner (Fig. 5g). Ki67-positive cells significantly reduced in Ti_3_C_2_ nanosheet-exposed GC-1 cells (Fig. 5h). These results strongly indicate that Ti_3_C_2_ nanosheet exposure inhibits the proliferation of mice testicular tissues and GC-1 cells.

### Ti_3_C_2_ nanosheets exposure induces cell cycle arrest in mouse testes and GC-1 cells

**Fig. 6:**
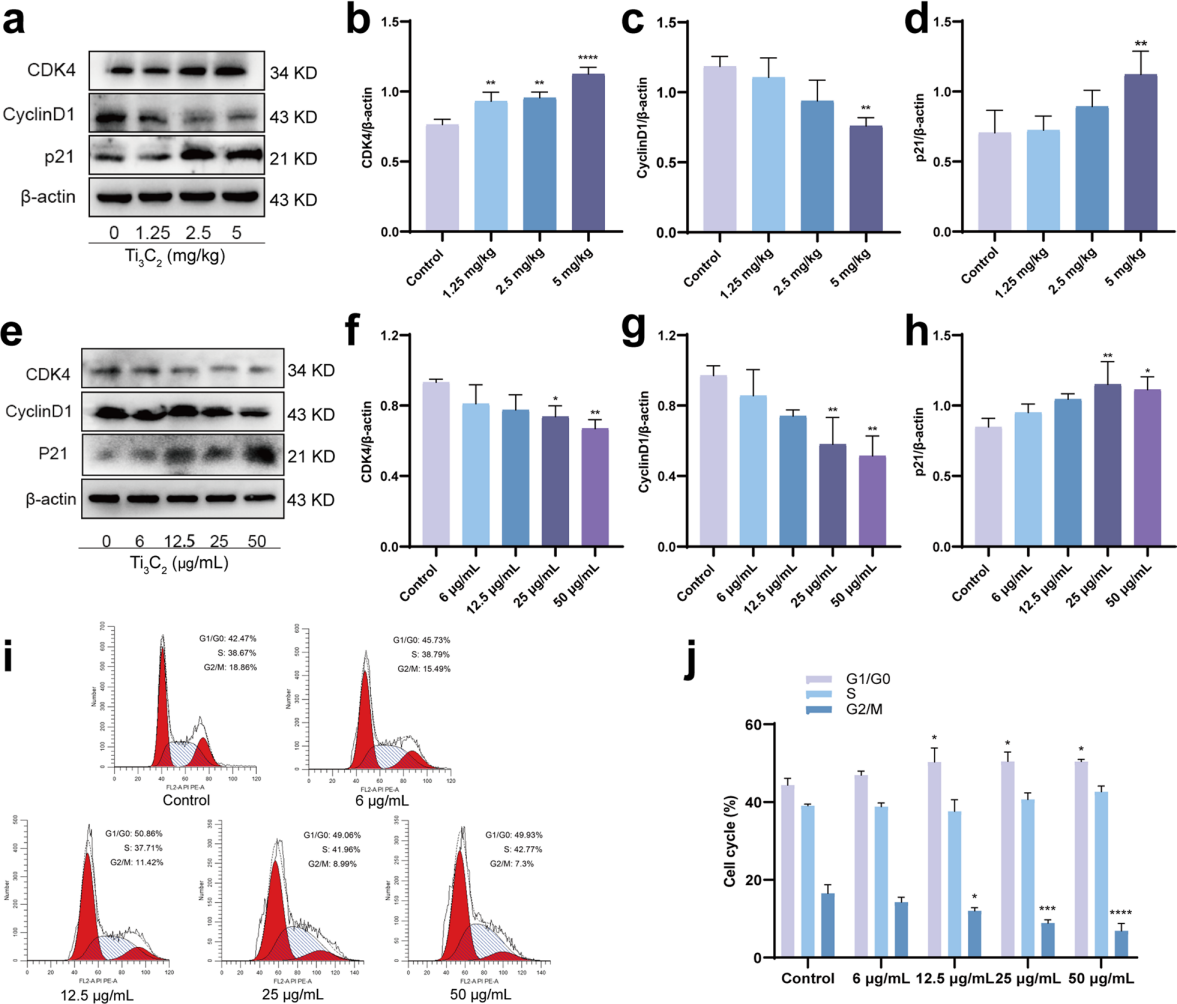
Ti_3_C_2_ nanosheets exposure induces cell cycle arrest in mouse testes and GC-1 cells. **a**–**d** Testicular G1/G0 phase associated proteins, (CDK4, CyclinD1, and p21) were detected using Western blotting. **e**–**h** Cellular G1/G0 phase associated proteins (CDK4, CyclinD1, and p21) were detected using Western blotting. **i**, **j** Cell cycle was measured by flow cytometry (n = 3). All data were expressed as means ± standard deviation (n = 3). *P < 0.05, **P < 0.01, ***P < 0.001, ****P < 0.0001, compared with the control group

G1/G0-related proteins were measured in Ti_3_C_2_ nanosheet-exposed mice testes to determine the effects of the exposure to these nanosheets on the cell cycle. CyclinD1 protein expression levels significantly decreased, while CDK4 and p21 protein expression levels significantly increased in the testes of Ti_3_C_2_ nanosheet-exposed mice (Fig. 6a–d). Compared with the control group, the results revealed that the protein expression levels of CDK4 and CyclinD1 were significantly lower, and p21 protein expression was increased in the GC-1 cells after Ti_3_C_2_ nanosheet exposure (Fig. 6e–h). Flow cytometry was used to detect the cell cycle of GC-1 cells after exposure to different concentrations of Ti_3_C_2_ nanosheets (0, 6, 12.5, 25, and 50 µg/mL) for 24 h. Compared to the control, the proportion of cells in the G2/M phase was reduced and that of cells in the G1/G0 phase was increased, whereas that of cells in the S phase did significantly change in the Ti_3_C_2_ nanosheet-exposed group (Fig. 6i, j). These results strongly indicate that Ti_3_C_2_ nanosheet exposure induced cell cycle arrest in GC-1 cells and mouse testes.

### Ti_3_C_2_ nanosheets exposure induces apoptosis in mouse testes and GC-1 cells

**Fig. 7:**
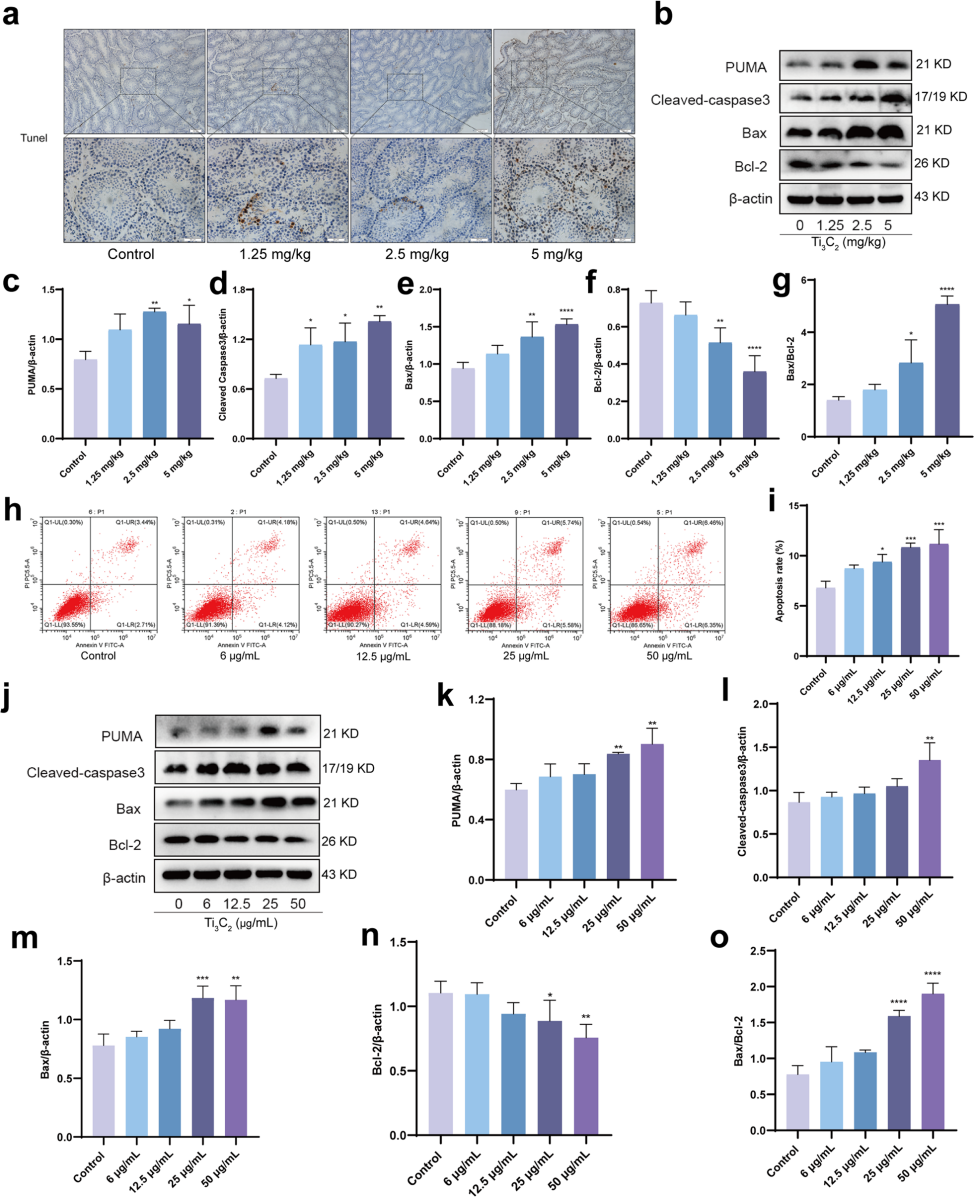
Ti_3_C_2_ nanosheets exposure induces apoptosis in mouse testes and GC-1 cells. **a** Testicular cross-sections were stained with TUNEL technique using Colorimetric TUNEL Apoptosis Assay Kit. Magnification at 100 × and 400 ×. **b–g** Testicular PUMA, Cleaved-caspase3, Bax, and Bcl-2 were detected using Western blotting. **h**, **i** Apoptosis was determined by flow cytometry using annexin V-FITC/PI staining. The apoptotic rate was calculated by the Annexin V-positive staining cells. **j**–**o** Cellular PUMA, Cleaved-caspase3, Bax and Bcl-2 were detected using Western blotting. All data were expressed as means ± standard deviation (n = 3). *P < 0.05, **P < 0.01, ***P < 0.001, ****P < 0.0001, compared with the control group

To further evaluate the effect of Ti_3_C_2_ nanosheets on spermatogenesis, we conducted TUNEL staining of testicular sections. As shown in Fig. 7a, the number of TUNEL-positive cells in the Ti_3_C_2_ nanosheet-exposed group significantly increased. Moreover, the expression levels of PUMA, Cleaved-caspase3 and Bax/Bcl-2 were remarkably higher in the Ti_3_C_2_ nanosheet-treated groups than that in the control group (Fig. 7b–g). In addition, flow cytometry showed that the apoptotic rate of GC-1 cells was significantly higher in Ti_3_C_2_ nanosheet-exposed cells than that in the control group (Fig. 7h, i). The expression levels of PUMA, Cleaved-caspase3, and Bax/Bcl-2 were remarkably higher in Ti_3_C_2_ nanosheet-treated GC-1 cells than that in the control group (Fig. 7j–o). These findings reveal that Ti_3_C_2_ nanosheet exposure induced apoptosis in mouse testes and GC-1 cells.

### ATM/p53 signaling pathway is involved in Ti_3_C_2_ nanosheets exposure-induced spermatogenesis disorder

**Fig. 8:**
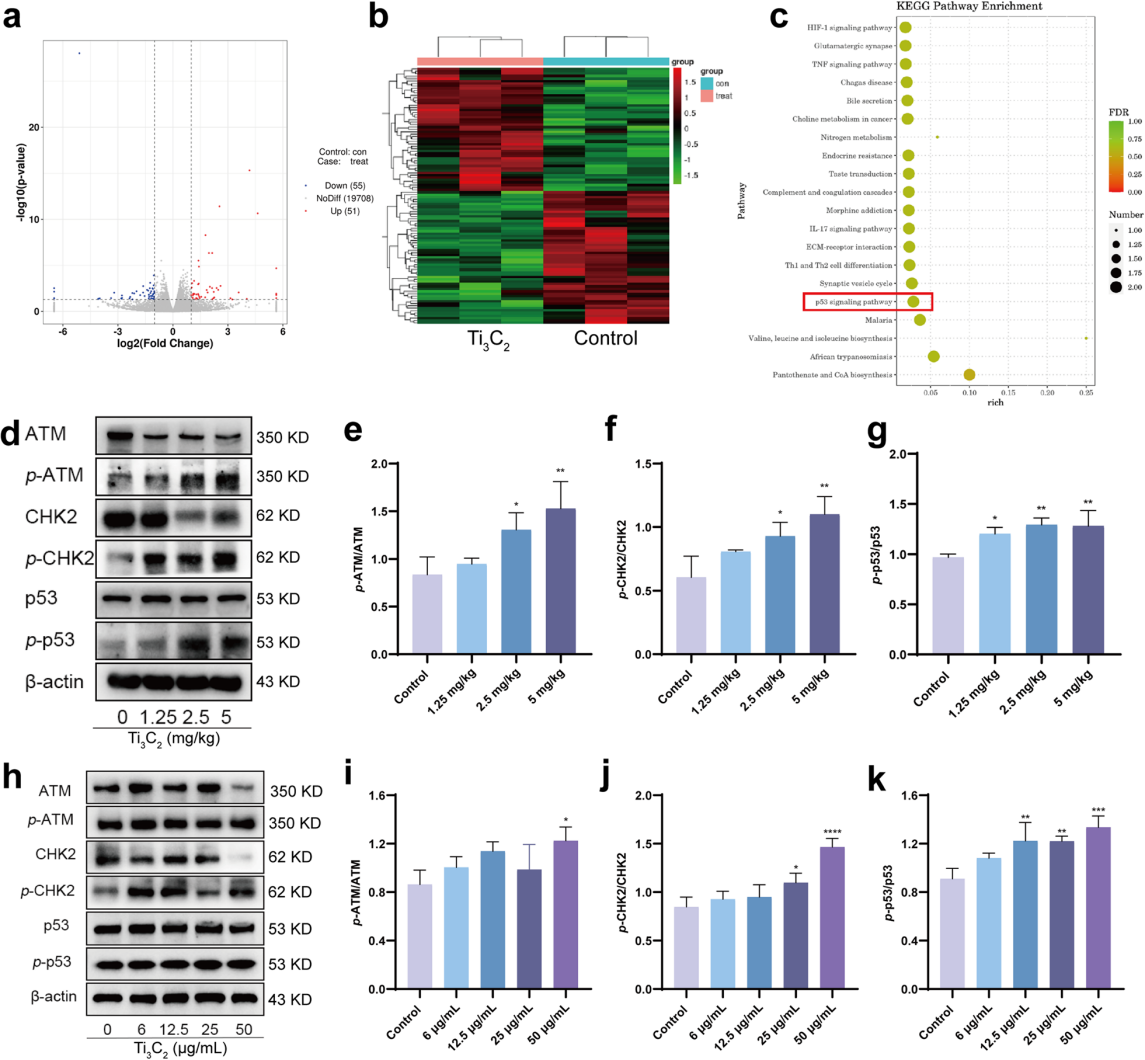
ATM/p53 signaling pathway is involved in Ti_3_C_2_ nanosheets exposure-induced spermatogenesis disorder. **a** Volcano plot of DEGs. **b** Heat map of DEGs in the samples. **c** KEGG pathway enrichment analysis of gene expression in testes. **d**–**g** Testicular ATM, *p*-ATM, CHK2, *p*-CHK2, p53, *p*-p53 were detected using Western blotting. **h**–**k** Cellular ATM, *p*-ATM, CHK2, *p*-CHK2, p53, *p*-p53 were detected using Western blotting. All data were expressed as means ± standard deviation (n = 3). *P < 0.05, **P < 0.01, ***P < 0.001, ****P < 0.0001, compared with the control group

To identify possible mechanisms of reduced spermatogonia proliferation and increased apoptosis due to Ti_3_C_2_ nanosheet exposure, we analyzed DEGs between Ti_3_C_2_ nanosheet-exposed and control tissues. The volcano map (Fig. 8a) and heat map (Fig. 8b) showed 106 differential genes between the Ti_3_C_2_ nanosheet-exposed and control groups (adjusted *P* value < 0.05), with 51 and 55 upregulated and downregulated genes in testicular tissue, respectively. KEGG pathway enrichment analysis was performed using these DEGs in mice testicular tissue. We found that the ATM/p53 signaling pathway were altered in enriched KEGG pathways (Fig. 8c). ATM/p53 is a key signaling in cell cycle checkpoint signaling. We explored the role of the ATM/p53 signaling pathway in Ti_3_C_2_ nanosheet-induced DNA damage. Western blotting was used to evaluate the expression of proteins related to the ATM/p53 signaling pathway. In mice testicular tissues and GC-1 cells, the phosphorylation levels of ATM, CHK2, and p53 were significantly elevated in Ti_3_C_2_ nanosheet-treated groups compared to the control group (Fig. 8d–k). These results indicate that the ATM/p53 signaling pathway was activated after Ti_3_C_2_ nanosheet exposure and mediates the DDR, cell cycle arrest and apoptosis.

## Discussion

Ti_3_C_2_ nanosheets are relatively new 2D nanomaterials that have increasingly gained scientific attention for various applications. Owing to their unique photothermal properties, they are used in biomedical applications [34, 35]. Thus, humans are usually exposed to these nanomaterials, and their effects on the reproductive system are of interest. Its effects on placental and ovarian functions have been previously reported by our group, and the present study was concerned with its effect on spermatogenesis.

The decrease in sperm number and increase in sperm malformation rate reflect the decline in male fertility. Research has shown a clear correlation between infertile men and low sperm count [36]. In the present study, we found that Ti_3_C_2_ nanosheets accumulated in the testes, and in vitro studies indicated that Ti_3_C_2_ nanosheets infiltrate GC-1 cells in a dose-dependent manner. These findings reveal that Ti_3_C_2_ nanosheets traverse the BTB, as with other nanoparticles [37, 38]. In addition, Ti_3_C_2_ nanosheets reduced the sperm count and increased sperm malformation in mice. Sperm formation is the main function of the testes and is dependent on their normal structure. It is known that exposure to environmental pollutants can induce histopathological changes in testes [6, 39]. The present study demonstrates that Ti_3_C_2_ nanosheets exposure can cause testicular damage, such as vacuoles in seminiferous tubules and disorganization of the germ cell layer. However, there were no significant changes in body weight gain or relative testicular weight in the mice. These results are in accordance with the findings of Adebayo et al. [31], who found that cerium oxide nanoparticles did not change the relative weight of the testis. This reveals that Ti_3_C_2_ nanosheets cause abnormalities in the structure of testicular tissue, an important reason for spermatogenesis disorders.

Several reports indicate that NPs are most frequently involved in induction of neurotoxicity, cytotoxicity and oxidative stress [40, 41]. Oxidative stress and inflammatory responses are of high importance as critical mechanisms underneath the above listed toxicities [42]. Normally, oxidative and antioxidant systems in the body are in a dynamic balance. However, a disruption of the balance produces oxidative stress, which mainly manifests as an imbalance between ROS and antioxidants [43]. MDA is a typical indicator of membrane lipid peroxidation. SOD functions as an antioxidant and protects the cells from oxidative damage. Excessive ROS causes excessive production of lipid radicals such as MDA [44]. NPs triggered ROS generation in extracellular and intracellular ways. It is confirmed that NPs can be engulfed by phagocytic cell through endocytosis or pinocytosis [42, 45]. Then the innate immune defense mechanisms were triggered by these NPs which were treated as foreign substances [46]. During this process, ROS are generated in form of superoxide and hydrogen peroxide, and served as key mediators driving oxidative stress cascades [47]. In this study, we observed that Ti_3_C_2_ nanosheets deposited in mouse testes or entered GC-1 cells, and we assumed Ti_3_C_2_ nanosheet exposure induced ROS production and oxidative stress through the above methods. Oxidative stress often induces cellular DNA strand damages via the oxidative DNA damages. An existed work showed some NPs like titanium dioxide can induce DNA damage through direct or indirect ways. The direct way is the interaction between NPs and DNA; the indirect way is excessive ROS and other toxic ions cause DNA damage [42]. In this study, we found that Ti_3_C_2_ nanosheet exposure induced oxidative stress and DNA damage in tissues and cells in vitro, and the mechanism of oxidative stress induction will be further explored.

Spermatogenesis is a unique male germ cell differentiation process and a unique cell cycle process, mainly through the proliferation of spermatogonia, meiosis of spermatocytes, and spermatocyte metamorphosis. Maintaining normal spermatogonia proliferation is important for sperm production [48]. Several studies have shown that nanoparticles or environmental pollutants can impair spermatogenesis by affecting germ cell proliferation and inducing apoptosis [49, 50]. To further reveal the cause of reduced spermatogenesis, we observed cell proliferation and apoptosis and found that Ti_3_C_2_ nanosheets inhibited proliferation and increased apoptosis in testicular tissues and GC-1 cells. This explains the previously observed decrease in sperm count. Cell cycle regulation is essential for maintaining genomic integrity against DNA damage and can provide more time for DNA repair to avoid replication and mutation. When the integrity of the cellular genome is challenged by extrinsic and intrinsic stresses, cells initiate the DNA damage response (DDR), in order to deliver intact genetic information to offspring [51]. p53 plays a crucial role in the DDR process. If the damaged DNA molecule recovers its structure, the cell enters the normal cell cycle.When DNA damage is constant, excessive DSBs trigger cell cycle arrest, leading to cell proliferation inhibition and irreversible apoptosis [52]. In Y. Liu’s research, the polystyrene nanoparticles (NH2ePS) caused a prolonged G1/G0 phase in the cell cycle and decreased the expression level of cyclin D and cyclin E, which implied DNA damage and the activation of checkpoint controls [53]. In this study, cell cycle arrest and the failure of DDR explain the proliferation inhibition and apoptosis of spermatogonia caused by Ti_3_C_2_ nanosheets.

Transcriptome sequencing suggested that Ti_3_C_2_ nanosheets could lead to significant changes in the ATM/p53 signaling pathway. ATM kinase is the most upstream DDR kinase in mammalian cells [54]. ATM is activated in response to DNA double-strand breaks (DSBs) [51]. ATM phosphorylates CHK2 on thr68, and CHK2 also causes cell cycle arrest by phosphorylating the tumor suppressor 53 (p53) [55]. *p53* is a transcription factor which involved in DNA repair, cell cycle arrest, and apoptosis. Active p53 promotes the transcription of *CDKN1A*, which encodes the cyclin-dependent kinase inhibitor p21[56]. p21 binds to the cell cycle protein, Cyclin D/Cdk4 in a complex, resulting in cell cycle arrest in the G1 phase [57]. A recent study showed that decabromodiphenyl ether (BDE-209) activates the ATM/p53 signaling pathway in mouse testes through oxidative stress-mediated DNA damage [49]. In the present study, the levels of phosphorylated ATM, CHK2, and p53 were remarkably elevated in mouse testes and GC-1 cells after Ti_3_C_2_ nanosheet exposure. The Ti_3_C_2_ nanosheet-exposed GC-1 cells were blocked in the G1/G0 phase, and this is the reason why we choose the ATM/p53 signaling as a possible mechanism for exploring the toxicity of Ti_3_C_2_ nanosheets. p53 activation also leads to the transcription of several pro-apoptotic genes including *Puma*, *BAX*, resulting in cell apoptosis if the damage is sustained [58]. Several studies have shown that Bcl-2 family and mitochondrial proteins are associated with p53-dependent apoptosis [59]. Following DNA damage, PUMA is triggered in a p53-dependent manner [60]. PUMA plays a core role in mitochondria-mediated cell death by reacting with all known members of the anti-apoptotic Bcl-2 family [61]. In the mitochondrial apoptotic pathway, an increase in the ratio of Bax to Bcl-2 activates caspase9. Upon activation, caspase9 activates caspase3, as a key initiator of apoptosis [30]. In the present study, Ti_3_C_2_ nanosheets promoted apoptosis in testicular tissue and GC-1 cells via the activation of the mitochondrial apoptotic pathway. These results and mechanisms well explained the reduced sperm count and increased sperm malformation rate caused by exposure to Ti_3_C_2_ nanosheet.

## Conclusions

Our results suggested that Ti_3_C_2_ nanosheet accumulated in the testis through the blood-testis barrier and occurred in GC-1 in vitro, which leaded to severe damage to the spermatogenic function of mice. We investigated the mechanism and found that exposure to Ti_3_C_2_ nanosheets leads to an increase of ROS, disrupting the balance of the oxidative and antioxidant system in the body and inducing oxidative stress. Oxidative stress causes DNA oxidative damage in spermatogonia, which is extremely unfavorable for germ cell to maintain the stability of genetic material. Meanwhile, p21 protein was up-regulated by mediating the activation of ATM/p53 signaling pathway, and the cell cycle arrested in G1/G0 phase for DDR. Persistent DNA damage causes *p53* to activate the transcription of pro-apoptotic factors, and the damaged spermatogonia goes to the fate of apoptosis. These links form a closed loop with the exposure of Ti_3_C_2_ nanosheets leading to a decrease in mice sperm quality. This study provides new experimental evidence on the mechanism of toxicity of Ti_3_C_2_ nanosheets on the male reproductive system and also some strategies for the safe application of the new two-dimensional nanomaterial Ti_3_C_2_ (MXene).

## Data Availability

All the original data are available upon reasonable request for correspondence authors.

## Abbreviations

2D: Two dimensional
EDCs: Endocrine-disrupting chemicals
ROS: Reactive oxygen species
TEM: Transmission electron microscopy
SEM: Scanning electron microscopy
AFM: Atomic force microscopy
IHC: Immunohistochemistry
IF: Immunofluorescence
MDA: Malondialdehyde
SOD: Superoxide dismutase
ELISA: Enzyme-linked immunosorbent assay
DCFH-DA: 2,7-Dichlorodihydrofluorescein diacetate
LMPA: Low melting point agarose
NMPA: Normal melting point agarose
NPs: Nanoparticles
CCK-F: Cell fluorescence counting kit
DEGs: Differentially expressed genes
WB: Western Blot
PVDF: Polyvinylidene fluoride
SD: Standard deviation
H&E: Hematoxylin and eosin
